# Simultaneous Assay of Dexchlorpheniramine Maleate, Betamethasone, and Sodium Benzoate in Syrup by a Reliable and Robust HPLC Method

**DOI:** 10.1155/2019/2952075

**Published:** 2019-11-29

**Authors:** Dinh Chi Le, Thi Duyen Ngo, Thi Huong Hoa Le

**Affiliations:** ^1^Department of Analytical Chemistry and Toxicology, Hanoi University of Pharmacy, Hanoi, Vietnam; ^2^National Institute of Drug Quality Control, Ministry of Health, Hanoi, Vietnam

## Abstract

The simultaneous determination of betamethasone, dexchlorpheniramine maleate, and sodium benzoate in pharmaceutical syrup was done by using a simple validated HPLC method. The chromatographic separation of the three analytes was done in a C18 column maintained at 25°C, using a mixture of acetonitrile and 0.02 M phosphate buffer solution pH 2.70 (35 : 65, v : v) as mobile phase. The isocratic elution was chosen with total flow rate of mobile phase maintained at 1.0 mL per minute. The analytes were detected by a UV-Vis detector set at 254 nm. Injection volume was set at 50 *μ*l. The method was fully validated in terms of specificity, linearity, precision, accuracy, and robustness according to requirements of current guidelines and was proven to be suitable for the intended application.

## 1. Introduction

Betamethasone is a synthetic glucocorticoid possessing anti-inflammatory [1] and antiallergic properties [2]. It works by affecting the synthesis of prostaglandin [3] and leukotriene [4]. Dexchlorpheniramine, usually used in form of maleate salt, is the pharmacologically active dextrorotatory enantiomer of chlorpheniramine [5], an antihistamine working on H1 receptors to reduce the allergic reactions [6]. Because betamethasone and dexchlorpheniramine produce their pharmacologic effects through different mechanisms, they can be used in combination in different dosage forms like tablets, oral solutions, or syrup to enhance the resulting therapeutic efficacy.

Besides the active principles, many pharmaceutical dosage forms also contain excipients for many purposes, including enhancing the efficacy of active principles (by ameliorating their solubility, by slowing down their deterioration, etc.) and assuring the efficiency and safety of the dosage form by prohibiting the development of micro-organism. These agents must be present in the pharmaceutical dosage forms at a proper level during the shelf life of these products to ensure their effectiveness. Therefore, the assay of solubility enhancers or antimicrobial preservatives, particularly the latter, is usually necessary in specification of pharmaceutical preparations, besides the assay of active principles.

Sodium benzoate can be used for both the abovementioned purposes: it can increase the solubility of active principle [7], and it can also be used as antimicrobial preservative [8, 9] to inhibit the development of micro-organism. So, the assay of sodium benzoate is required in these cases for quality control purpose.

Betamethasone and dexchlorpheniramine maleate were determined separately in bulk active compound and pharmaceutical dosage forms by HPLC in C18 column [10–13]. Betamethasone and dexchlorpheniramine maleate were simultaneously determined by UV-Vis spectrometry [14] and high-performance thin-layer chromatography [15]. The assay of sodium benzoate can be done by volumetric titration [7]. However, for the simultaneous assay of sodium benzoate and other analytes, HPLC in C18 column was the most common choice of analytical technique [8, 9, 16]. Up to now, no method has yet been published for simultaneous determination of betamethasone, dexchlorpheniramine maleate, and sodium benzoate in pharmaceutical dosage forms.

To assure the reliability of analytical results, any method intended for the assay of active principle(s) and other components, such as preservative(s), in pharmaceutical dosage forms must be able to satisfy suitable performance levels, such as those required by AOAC International for quantitative method [17] and must be able to provide obligated validation data to authorities according to guidelines on analytical method validation, such as those issued by ICH [18] or FDA [19].

In the current guideline “Validation of Analytical Procedures: Text and Methodology Q2 (R1)” of ICH, assay tests for drug substances and drug product must be validated in terms of specificity, precision, accuracy, linearity, and range [18]. The validation of an analytical procedure ensures the reliability and reproducibility of results obtained from the applied analytical technique and the particular analytical conditions of the method. The validation report of all analytical methods must be submitted to the regulation agency as an integral part of the technical document for the registration of pharmaceuticals for human use.

In this study, an HPLC method using C18 column was developed and validated for simultaneous assay of betamethasone, dexchlorpheniramine maleate, and sodium benzoate in syrup.

## 2. Materials and Methods

### 2.1. Instrumentation

The method was developed and validated on a Shimadzu LC-20AT HPLC system (Shimadzu, Kyoto, Japan) consisting of a pump (model LC-20AD), a degasser (model DGU-20A5), a PDA detector (model SPD-M20A), an autosampler (model SIL-20ACHT), and a control module (model CBM-20Alite). This system used LC solution software version 1.25 SP4 for data processing and evaluation. Analytical column was a Luna C18 column (250 × 4.6 mm, 5 *μ*m) of Phenomenex (Torrance, CA, USA).

### 2.2. Chemicals and Reagents

Reference substances of betamethasone (purity 100.4%) and sodium benzoate (purity 98.6%) were established at National Institute of Drug Quality Control (Hanoi, Vietnam); reference substance of dexchlorpheniramine maleate (purity 99.9%) was purchased from Institute of Drug Quality Control of Ho Chi Minh City (Ho Chi Minh City, Vietnam). Xinfadro syrup (containing 3.0 mg of betamethasone, 24.0 mg of dexchlorpheniramine maleate, and 120.0 mg of sodium benzoate per 60 mL of syrup) was purchased from market. A placebo mixture consisting of citric acid, sodium citrate, vanillin, sorbitol, ethanol, refined sugar, and water was prepared from the information provided in the label of syrup bottle to be used in method validation steps. Acetonitrile HPLC grade, methanol HPLC grade, orthophosphoric acid PA grade, potassium dihydrogen phosphate PA grade, and triethylamine PA grade were purchased form Merck Vietnam (Ho Chi Minh City, Vietnam).

### 2.3. Chromatographic Conditions

Mobile phase was a mixture of acetonitrile and 0.02 M phosphate buffer solution pH 2.7 (35 : 65, v : v). The 0.02 M phosphate buffer solution pH 2.7 was prepared by dissolving 2.72 g of potassium dihydrogen phosphate and 3 mL of triethylamine in 900 mL of water, adjusting the pH to 2.7 ± 0.1 by orthophosphoric acid, adding water to make 1000 mL, mixing the solution well, filtering it through 0.45 *μ*m membrane filter, and degassing it by sonication for 15 minutes before using it. The flow rate of mobile phase was maintained at 1.0 mL/min. The analysis was carried out on an Shimadzu LC-20AT series HPLC system equipped with a PDA detector set at 254 nm for recording chromatograms. The chromatographic separation was conducted on a Luna C18 column (250 × 4.6 mm, 5 *μ*m) maintained at 25°C. The injection volume was 50 *μ*l.

### 2.4. Preparation of Standard Solution

Stock standard solutions of betamethasone (1.0 mg/mL), dexchlorpheniramine maleate (1.0 mg/mL), and sodium benzoate (5.0 mg/mL) were prepared by dissolving an accurately weighed quantity of corresponding reference standards using mobile phase as diluents. Working mixed standard solutions were prepared by accurately diluting stock standard solutions to the intended concentration with the same diluents. Standard solutions were filtered through 0.45 *μ*m membrane filter before being used for chromatographic analysis.

### 2.5. Preparation of Sample Solution and Placebo Solution

To prepare sample solution, an amount of syrup equivalent to about 0.25 mg of betamethasone was accurately weighed into a 25 mL volumetric flask and was diluted to volume with mobile phase as diluent. This solution was filtered through 0.45 *μ*m membrane filter before being used for chromatographic analysis.

For method validation, placebo solution was prepared by weighing accurately a quantity of placebo mixture (as described in 2.2) equivalent to the amount of syrup used to prepare sample solution and diluted afterward as with the sample solution.

### 2.6. Method Validation

To assure the suitability of the method for simultaneous assay of dexchlorpheniramine, betamethasone, and sodium benzoate in syrup, it was validated in accordance with the current guideline of ICH [18] in the following criteria.

#### 2.6.1. Specificity

In the case of HPLC method, the specificity is assured by the complete separation of analytes of interest from other components in the sample matrix [16]. To evaluate the capacity of the developed method to yield well-separated peaks corresponding to dexchlorpheniramine, sodium benzoate, and betamethasone, mixed standard solution of these analytes, sample, placebo, and blank solution were injected separately at the same volume (50 *μ*L) into the chromatographic system.

#### 2.6.2. Linearity and Range

According to the guideline of ICH [18], an assay method must maintain linear relation between the concentration of analyte(s) and the intensity of response (i.e., peak area for HPLC method) within a certain range around the target concentration (at least from 80% to 120% of target concentration). In this study, the target concentration was about 10.0 *μ*g/mL for betamethasone, 80.0 *μ*g/mL for dexchlorpheniramine, and 400.0 *μ*g/mL for sodium benzoate. Accordingly, mixed standard solutions containing exact concentrations of betamethasone, dexchlorpheniramine maleate, and sodium benzoate at different levels of betamethasone (6.0, 8.0, 10.0, 12.0, 13.9, and 15.9 *μ*g/mL), dexchlorpheniramine maleate (48.2, 64.3, 80.4, 96.5, 112.6, and 128.6 *μ*g/mL), and sodium benzoate (240.2, 320.3, 400.4, 480.5, 560.6, and 640.6 *μ*g/mL) were prepared, corresponding to 60%, 80%, 100%, 120%, 140%, and 160% of target concentration, respectively. Three injections of mixed standard solution at each concentration were executed and calibration curve for each analyte was established between the standard concentration and average peak area. The significance of the linearity of each calibration curve was assessed by one-way ANOVA (the linearity is significant if *P* < 0.05 or Sig. <0.05 in expression of SPSS 16.0 software) [16].

#### 2.6.3. Sensitivity

For HPLC methods, generally, the sensitivity is assessed by measuring the signal-to-noise ratio between the peak height of analyte and the variation of the neighboring baseline in chromatograms. The concentrations of analyte giving a signal-to-noise ratio about 3 : 1 and about 10 : 1, respectively, are considered as the lowest detectable level or limit of detection (LOD) and the lowest quantifiable level or limit of quantitation (LOQ) [16, 20]. The LOD and LOQ of betamethasone, dexchlorpheniramine maleate, and sodium benzoate were determined by analyzing solutions containing these substances at different concentrations and measuring the signal-to-noise ratio for each analyte.

#### 2.6.4. Accuracy

According to the current guideline [18] and the previously published work [16], the accuracy for an assay method must be validated by recovery studies at at least three concentrations of each analyte within the range from 80% to 120% of target concentration. Therefore, to evaluate the accuracy in quantitative determination of each analyte, exact quantities of reference substances of betamethasone, dexchlorpheniramine maleate, and sodium benzoate were mixed with placebo matrix in such a way that the spiked samples, after preparation process, yielded solutions containing each analyte at three concentration levels, corresponding to 80%, 100%, and 120% of target concentration, i.e., about 0.008, 0.010, and 0.012 mg/mL with betamethasone; 0.064, 0.080, and 0.096 mg/mL with dexchlorpheniramine maleate; and about 0.320, 0.400, and 0.480 mg/mL with sodium benzoate. At each concentration level, three samples were prepared and analyzed to obtain the percentage recovery of each analyte and the RSD for variation of recovery rate at each concentration level.

#### 2.6.5. Precision

The precision of chromatographic system, or system suitability, was validated by estimating the variation of peak performance of each analyte after six repetitive injections of mixed standard solution of dexchlorpheniramine, betamethasone, and sodium benzoate at 100% of target concentrations [16, 18, 20].

The method's precision, including repeatability (intraday precision) and intermediate precision (interday precision), was determined by calculating the variation of quantitative result obtained from six independent analyses of sample solutions containing dexchlorpheniramine, betamethasone, and sodium benzoate at approximately 100% of target concentration on the same day and on two different days, respectively.

#### 2.6.6. Range

Range of concentrations of each analyte where the accuracy and precision of quantitative analysis are assured must be at least from 80% to 120% of the target concentration of each analyte [18, 19]. This requirement was validated simultaneously with the accuracy of the method as mentioned above.

#### 2.6.7. Robustness

The current ICH guideline [18] does not obligate the robustness on validation of assay method but welcomes any attempt to confirm the robustness of an analytical method, particularly a quantitative one. In this study, following small and deliberate changes on HPLC conditions was applied to assess the impact on analytical results:

- Flow rate: ±0.2 mL/min
- Percentage of 0.02 M phosphate buffer solution in mobile phase: ±1%
- pH of the 0.02 M phosphate buffer solution: ±0.5 pH units

At each condition, a mixed standard solution of dexchlorpheniramine maleate, sodium benzoate, and betamethasone at 100% of target concentration and three sample solutions at approximately 100% target concentration were prepared and injected into chromatography system. The robustness of the method was verified by investigating the variation in peak area of each analyte in repetitive analysis of standard solution and the variation in the content of each analyte found in sample solutions [16, 20, 21].

#### 2.6.8. Stability of Analytical Solution

Although the standard and sample solutions were analyzed immediately after preparation, there is always a delay time during which these solutions waited to be analyzed in the autosampler tray. Therefore, their stability was investigated by analyzing the standard and sample preparations at 0 h and after one day of cool storage (at 10°C in refrigerator) and at 25°C. For each solution, three injections were executed at each time, and the stability of analytical solutions was evaluated from the variation of average peak area and RSD value of peak area among repeated injections.

### 2.7. Data Processing

IBM SPSS software (version 16.0) (IBM, Armonk, NY, USA) was used for statistical analysis of analytical results.

## 3. Results and Discussion

### 3.1. Method Development and Optimization

The objective of this method is to provide a chromatographic solution that permits simultaneous assay of the two active principles dexchlorpheniramine maleate and betamethasone and the preservative sodium benzoate in syrup. From the information gathered after our bibliographic research, an analytical column with end-capped octadecylsilyl stationary phase, the Luna C18 column, the stationary phase was selected for method development.

To obtain chromatographic conditions suitable for the intended application of the method, preliminary trials were carried out. The results obtained from these trials were summarized in Table 1. They pointed out that the use of acetonitrile as the organic component in mobile phase would give better peak shape for dexchlorpheniramine maleate and sodium benzoate and give shorter analysis time than methanol when used at the same percentage in mobile phase. Preliminary test also found that phosphate buffer gives better peak shapes for analytes and better resolution between analytes and other matrix components. From these results, Luna C18 column with acetonitrile and 0.02 M phosphate buffer solution pH 2.7 (35 :65, v : v) in isocratic elution mode was selected for the final method.

### 3.2. Method Validation

#### 3.2.1. Specificity

**Figure 1:**
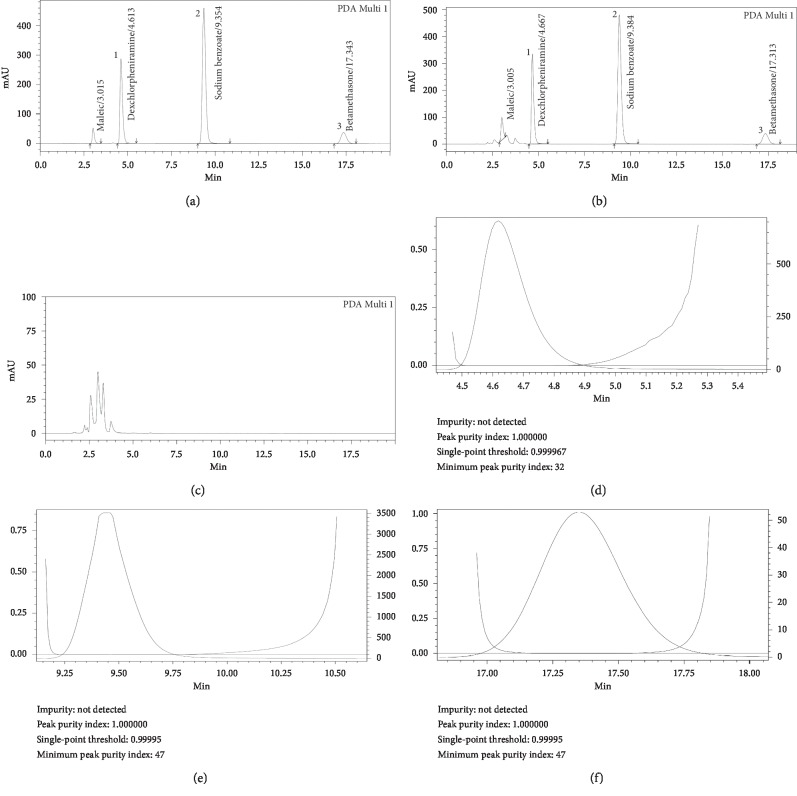
Chromatogram of mix standard solution (a), Xifapro sample solution (b), placebo (c) and peak purity of analytes (peak of dexchlorpheniramine (d), peak of sodium benzoate (e) and peak of betamethasone (f)). 1, peak of dexchlorpheniramine, 2, peak of sodium benzoate, 3, peak of betamethasone.

To evaluate the specificity of the method, blank solution, placebo solution, standard solution, and sample solution (containing betamethasone, dexchlorpheniramine maleate, and sodium benzoate at target concentrations, i.e., 0.010 mg/mL, 0.080, and 0.400 mg/mL, respectively) were injected separately into HPLC system, and the chromatogram results are shown in Figures 1(a)–1(c). Betamethasone, dexchlorpheniramine maleate, and sodium benzoate were eluted into 3 well-separated peaks, and purity analysis (Figures 1(d)–1(f)) confirmed that there was no coeluted element at the retention times of any analyte. Therefore, the chromatographic separation was capable of isolating each of the analytes of interest and permitting their specific analysis without interference from other components of the sample matrix.

#### 3.2.2. Linearity

**Figure 2:**
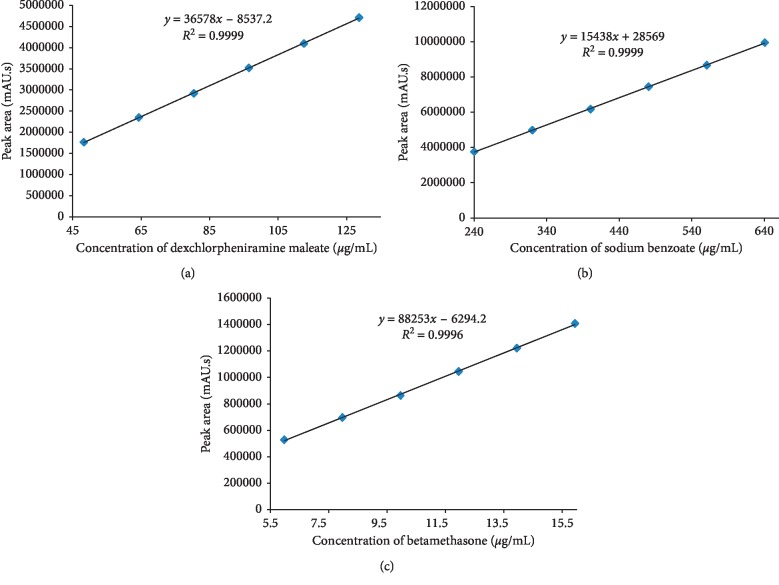
Calibration curves of dexchlorpheniramine maleate (a), sodium benzoate (b), and betamethasone (c).

The mean peak area of each analyte obtained from the chromatogram of mixed standard solution was plotted against corresponding concentration to establish the calibration line. The summarized graphs (Figure 2) revealed linearity over the concentration range of 6.0–15.9 *μ*g/mL for betamethasone, of 48.2–128.6 *μ*g/mL for dexchlorpheniramine maleate, and of 240.2–640.6 *μ*g/mL for sodium benzoate. From the regression analysis, the linear equation was obtained: *y* = 88252*x* − 6294 for betamethasone, *y* = 36578*x* − 8537 for dexchlorpheniramine maleate, and *y* = 15438*x* + 28569 for sodium benzoate, and the coefficient of determination *R*-square was 0.999 for all the three analytes. ANOVA analysis for all analytes (Tables 2–4) confirmed the statistical significance of the linear regression model in predicting the outcome variable (*P* < 0.05).

#### 3.2.3. Limit of Detection (LOD) and Limit of Quantification (LOQ)

For betamethasone, the concentration of injected solution at LOD and LOQ was 2.0 *μ*g/mL and 6.0 *μ*g/mL, equivalent to injected quantity of betamethasone of 0.10 *μ*g and 0.30 *μ*g, respectively. For dexchlorpheniramine maleate, the concentration of injected solution at LOD and LOQ was 16.0 *μ*g/mL and 48.0 *μ*g/mL, equivalent to injected quantity of potassium guaiacolsulfonate of 0.80 *μ*g and 2.40 *μ*g, respectively. For sodium benzoate, the concentration of injected solution at LOD and LOQ was 80.0 *μ*g/mL and 240.0 *μ*g/mL, equivalent to injected quantity of sodium benzoate of 4.00 *μ*g and 12.00 *μ*g, respectively.

#### 3.2.4. Accuracy

The ICH guideline [18] requires that recovery rate for assay method must fall between 98.0% to 102.0% of the true concentration and variation of recovery rate at one concentration level in terms of RSD must not exceed 2.0%. The recovery studies with dexchlorpheniramine, betamethasone, and sodium benzoate, summarized in Table 5, showed recovery rate from 99.8% to 102.0% at all three levels for all analytes and at RSD values at each level for each analyte varying from 0.1 to 0.6%, within the limits recommended by ICH. So, the method was of acceptable accuracy for simultaneous assay of dexchlorpheniramine, betamethasone, and sodium benzoate in syrup.

#### 3.2.5. Precision

The system precision, or system suitability, of the method was revealed by the peak performance for each analyte. For dexchlorpheniramine, betamethasone, and sodium benzoate, the variations of peak properties (retention time, area, tailing factor, and number of theoretical plates) in terms of RSD were all under 2.0%, as presented in Table 6, with number of theoretical plates higher than 1000 for the peaks of three analytes.

The repeatability and the intermediate precision of the method were assessed by the variations in assay results of dexchlorpheniramine, betamethasone, and sodium benzoate. As presented in Table 7, these variations, both intraday (repeatability) and interday (intermediate precision), were less than 2.0% in terms of RSD for all analytes.

Therefore, in terms of system precision and method precision, the method was suitably precise for simultaneous assay of dexchlorpheniramine, betamethasone, and sodium benzoate in syrup.

#### 3.2.6. Range

As discussed in Section 3.2.4 and summarized in Table 5, the range from 80% to 120% of target concentration for dexchlorpheniramine, betamethasone, and sodium benzoate assured the accuracy and precision of assay results for these analytes.

#### 3.2.7. Robustness

After implementing deliberate minor changes as mentioned in Section 2.6.7, the peak area and assay results for each analyte obtained at each modified condition were presented in Table 8. For all applied changes, variation of peak area for all analytes was small (RSD less than 2.0%) and good separation was achieved for each analyte. The contents of betamethasone, dexchlorpheniramine maleate, and sodium benzoate found in sample were not varied significantly when any change described in Section 2.6.7 was implemented, as one-way ANOVA analysis found *F* < *F*_inscrit_ for both analytes (as shown in Table 9).

#### 3.2.8. Solution Stability

The percentage of recovery was within the range of 98.0% to 102.0% and RSD was not more than 2.0%, indicating a good stability of the sample and standard solutions for 24 hr at both conditions, as shown in Table 10. These results proved that both analytes were stable in sample and standard solutions prepared as described in 2.4 and 2.5, and the preparation procedure for sample and standard solution was suitable for intended application of the method.

## 4. Conclusion

In this paper, the development and validation of an HPLC method for simultaneous assay of dexchlorpheniramine maleate, sodium benzoate, and betamethasone in syrup have been discussed. The method has been proven to be able to quantify these three analytes specifically, without interference from sample matrix. The reliability and robustness of the method were also assured by validation results, demonstrating its suitability for intended application.

## Figures and Tables

**Table 1:** Results of preliminary optimization.

Column	Mobile phase	Elution mode	Flow rate	Observation	Result
Phenomenex C18	Methanol—phosphate buffer 0.02 M pH 2.7 (40 : 60, v : v)	Isocratic	1.0 mL/min	Poor peak shapes for dexchlorpheniramine maleate and sodium benzoate, retention too long for betamethasone (more than 20 minutes), betamethasone still partially coeluted with a matrix peak	Rejected
Phenomenex C18	Acetonitril—phosphate buffer 0.02 M pH 2.7 (40 : 60, v : v)	Isocratic	1.0 mL/min	Better peak shape for dexchlorpheniramine maleate and sodium benzoate, shorter analysis time, but betamethasone still partially coeluted with a matrix peak	Rejected
Phenomenex C18	Acetonitril—acetate buffer 0.025 M pH 3.0 (40 : 60, v : v)	Isocratic	1.0 mL/min	Bethamethasone was resolved from matrix peak but peak shape was poor and peak response unstable	Rejected
Phenomenex C18	Acetonitril—phosphate buffer 0.02 M pH 2.7 (35 : 65, v : v)	Isocratic	1.0 mL/min	Good peak shape for all three analytes and all three analytes were completely separated from matrix components	Accepted

**Table 2:** Results of ANOVA analysis for calibration curve of dexchlorpheniramine maleate.

Model		Sum of squares	d*f*	Mean square	*F*	Sig.
1	Regression	6,054E12	1	6,054E12	4,306E4	000^a^
	Residual	5,624E8	4	1,406E8		
	Total	6,055E12	5			

^a^Predictors (constant), dexchlorpheniramine_concentration. ^b^Dependent variable, dexchlorpheniramine_peak_area.

**Table 3:** Results of ANOVA analysis for calibration curve of sodium benzoate.

Model		Sum of squares	d*f*	Mean square	*F*	Sig.
1	Regression	2,675E13	1	2,675E13	3,571E4	000^a^
	Residual	2,996E9	4	7,490E8		
	Total	2,675E13	5			

^a^Predictors (constant), sodium_benzoate_concentration. ^b^Dependent variable, sodium_benzoate_peak_area.

**Table 4:** Results of ANOVA analysis for calibration curve of betamethasone.

Model		Sum of squares	d*f*	Mean square	*F*	Sig.
1	Regression	5,408E11	1	5,408E11	1,014E4	000^a^
	Residual	2,134E8	4	5,335E7		
	Total	5,411E11	5			

^a^Predictors (constant), betamethasone_concentration. ^b^Dependent variable, betamethasone_peak_area.

**Table 5:** Results of accuracy.

Spiked level (%)	Replicate number	Dexchlorpheniramine maleate			Sodium benzoate			Betamethasone		
		Spiked amount of standard (mg)	Peak area (mAU·s)	Recovery (%)	Spiked amount of standard (mg)	Peak area (mAU·s)	Recovery (%)	Spiked amount of standard (mg)	Peak area (mAU·s)	Recovery (%)
80%	1	1.606	2326791	99.7	7.895	5013454	101.6	0.199	695365	100.5
	2	1.606	2334539	100.0	7.895	4991733	101.2	0.199	703190	101.6
	3	1.606	2327665	99.7	7.895	5017325	101.7	0.199	696949	100.7
	Mean			99.8			101.5			100.9
	RSD (%)			0.2			0.3			0.6

100%	1	2.008	2890627	99.1	9.869	6225259	101.0	0.249	867571	100.3
	2	2.008	2899677	99.4	9.869	6238642	101.2	0.249	871281	100.8
	3	2.008	2885840	98.9	9.869	6216278	100.8	0.249	863282	99.8
	Mean			99.1			101.0			100.3
	RSD (%)			0.2			0.2			0.5

120%	1	2.409	3480064	99.4	11.843	7478069	101.1	0.298	1048690	101.1
	2	2.409	3469911	99.1	11.843	7470749	101.0	0.298	1047360	100.9
	3	2.409	3476467	99.3	11.843	7477084	101.1	0.298	1047448	100.9
	Mean			99.3			101.1			101.0
	RSD (%)			0.1			0.1			0.1

**Table 6:** Results of system precision for betamethasone and dexchlorpheniramine maleate.

No. of injection	Retention time (minutes)	Peak area (mAu·s)	Asymmetry of peak	Number of theoretical plates	Resolution
*Dexchlorpheniramine maleate*					
1	4.721	2919244	1.3	3962	5.2
2	4.708	2920606	1.3	3940	5.2
3	4.703	2922790	1.3	3932	5.2
4	4.689	2915735	1.3	3909	5.2
5	4.689	2913275	1.3	3909	5.2
6	4.689	2910823	1.3	3909	5.2
Average	4.700	2917079	1.3	3927	5.2
RSD (%)	**0.3**	**0.2**	**0.1**	**0.6**	**0.3**

*Sodium benzoate*					
1	9.410	6172807	1.3	2891	9.4
2	9.413	6173718	1.3	2893	9.4
3	9.415	6171398	1.3	2894	9.4
4	9.397	6162984	1.3	2883	9.4
5	9.386	6161866	1.3	2877	9.4
6	9.377	6150875	1.3	2871	9.4
Average	9.400	6165608	1.3	2885	9.4
RSD (%)	**0.2**	**0.1**	**0.1**	**0.3**	**0.2**

*Betamethasone*					
1	17.917	863391	1.1	8025	11.3
2	17.895	868096	1.1	8006	11.3
3	17.869	868453	1.1	7983	11.3
4	17.818	860990	1.1	7937	11.2
5	17.787	864537	1.1	7909	11.2
6	17.757	863288	1.1	7883	11.2
Average	17.841	864793	1.1	7957	11.3
RSD (%)	**0.4**	**0.3**	**0.1**	**0.7**	**0.6**

**Table 7:** Results of repeatability and intermediate precision.

No. of sample solution	Sample weight (g) Density: 1.095 g/mL	Content of dexchlorpheniramine maleate in syrup (%, comparing to labeled amount)	Content of sodium benzoate in syrup (%, comparing to labeled amount)	Content of betamethasone in syrup (%, comparing to labeled amount)
*Day 1*, *analyst 1*				
1	5.4324	101.0	101.2	101.5
2	5.4630	100.4	100.5	101.3
3	5.3981	100.7	101.1	101.6
4	5.5087	99.0	99.2	99.8
5	5.4672	100.0	100.2	100.2
6	5.5064	98.6	98.8	99.5
Average (1–6)		99.9	100.2	100.7
RSD (%) (1–6)		**1.0**	**1.0**	**0.9**

*Day 2*, *analyst 2*				
7	5.5623	99.6	101.2	101.5
8	5.4786	101.4	100.5	101.3
9	5.5368	100.2	101.1	101.6
10	5.4361	101.7	99.2	99.8
11	5.5698	100.1	100.2	100.2
12	5.5345	100.1	98.8	99.5
Average (1–12)		100.2	99.5	101.2
RSD (%) (1–12)		**0.9**	**1.1**	**1.0**

Results obtained in day 1 by analyst 1 (sample no. 1–6) were used for evaluating repeatability and those obtained in day 1 and day 2 (sample no. 1–12) were used together for evaluating intermediate precision.

**Table 8:** Results of robustness.

Variation	Specific condition	Dexchlorpheniramine maleate		Sodium benzoate		Betamethasone	
		RSD (%) for peak area	RSD (%) for content in syrup	RSD (%) for peak area	RSD (%) for content in syrup	RSD (%) for peak area	RSD (%) for content in syrup
Flow rate (mL/min)	0.8	0.1	0.4	0.1	0.4	0.3	0.4
	1.0 (normal)	0.2	0.6	0.2	0.6	0.4	0.5
	1.2	0.4	0.4	0.1	0.9	0.3	0.8

pH of 0.02 M phosphate buffer solution	2.65	0.2	0.6	0.2	0.6	0.2	0.6
	2.70 (normal)	0.1	0.5	0.3	0.9	0.4	0.3
	2.75	0.3	0.3	0.5	0.9	0.4	0.5

Percentage of 0.02 M phosphate buffer in mobile phase	64	0.1	0.5	0.2	0.9	0.2	0.5
	65 (normal)	0.1	0.6	0.3	0.5	0.3	0.7
	66	0.1	0.4	0.2	1.1	0.5	0.4

Wavelength of detector	252 nm	0.5	0.4	0.2	0.5	0.2	0.5
	254 nm (normal)	0.1	0.3	0.3	0.5	0.3	0.4
	256 nm	1.0	0.4	0.4	0.9	0.2	0.5

**Table 9:** Results of ANOVA analysis for content of betamethasone, dexchlorpheniramine maleate and sodium benzoate.

		Sum of squares	d*f*	Mean square	*F*	Sig.
Dexchlorpheniramine_content	Between groups	976	11	089	460	910
	Within groups	4,633	24	193		
	Total	5,610	35			

Sodium_benzoate_content	Between groups	2,014	11	183	317	974
	Within groups	13,853	24	577		
	Total	15,868	35			

Betamethasone_content	Between groups	1,123	11	102	403	941
	Within groups	6,073	24	253		
	Total	7,196	35			

**Table 10:** Results of stability studies.

Studies	Average retention time (minutes)	Average peak area (mAu.s)	Average asymmetry of peak	Average number of theoretical plate	RSD (%) of peak area	Recovery (%)
Dexchlorpheniramine maleate						
Standard solution						
0 h	4.698	2921785	1.3	3924	0.1	—
24 h at refrigerator	4.702	2917643	1.3	3930	0.1	99.9
24 h at 25°C	4.691	2914786	1.3	3912	0.2	99.8
Sample solution						
0 h	4.705	2917613	1.3	3935	0.2	—
24 h at refrigerator	4.695	2914534	1.3	3919	0.1	99.9
24 h at 25°C	4.701	2912677	1.3	3929	0.4	99.8

Sodium benzoate						
Standard solution						
0 h	9.403	6169304	1.3	2887	0.1	—
24 h at refrigerator	9.391	6167025	1.3	2880	0.1	100.0
24 h at 25°C	9.415	6149723	1.3	2894	0.1	99.7
Sample solution						
0 h	9.411	6170542	1.3	2892	0.2	—
24 h at refrigerator	9.396	6165339	1.3	2883	0.1	99.9
24 h at 25°C	9.409	6148732	1.3	2891	0.2	99.6

Betamethasone						
Standard solution						
0 h	17.807	862754	1.1	7927	0.1	—
24 h at refrigerator	17.789	861859	1.1	7911	0.1	99.9
24 h at 25°C	17.814	860934	1.1	7933	0.3	99.8
Sample solution						
0 h	17.792	861055	1.1	7914	0.1	—
24 h at refrigerator	17.801	859823	1.1	7922	0.2	99.9
24 h at 25°C	17.797	858506	1.1	7918	0.2	99.7

## Data Availability

The data used to support the findings of this study are available from corresponding author (hoalethihuong@gmail.com) upon request.

## References

[1] Kulkarni P. S., Bhattacherjee P., Eakins K. E., Srinivasan B. D. (1981). Anti-inflammatory effects of betamethasone phosphate, dexamethasone phosphate and indomethacin on rabbit ocular inflammation induced by bovine serum albumin. *Current Eye Research*.

[2] Grönneberg R., Strandberg K., Stålenheim G., Zetterström O. (1981). Effect in man of anti-allergic drugs on the immediate and late phase cutaneous allergic reactions induced by anti-IgE. *Allergy*.

[3] Lewis G. D., Campbell W. B., Johnson A. R. (1986). Inhibition of prostaglandin synthesis by glucocorticoids in human endothelial cells. *Endocrinology*.

[4] Zicari A., Ticconi C., Pontieri G., Loyola G., Piccione E. (1997). Effects of glucocorticoids and progesterone on prostaglandin E2 and leukotriene B4 release by human fetal membranes at term gestation. *Prostaglandins*.

[5] Yasuda S. U., Zannikos P., Young A. E., Fried K. M., Wainer I. W., Woosley R. L. (2002). The roles of CYP2D6 and stereoselectivity in the clinical pharmacokinetics of chlorpheniramine. *British Journal of Clinical Pharmacology*.

[6] Kirkegaard J., Secher C., Mygind N. (1982). Effect of the H_1_ Antihistamine chlorpheniramine maleate on histamine-induced symptoms in the human conjunctiva. *Allergy*.

[7] The United States Pharmacopoeial Convention (2017). *Caffeine and Sodium Benzoate Injection USP Monograph USP40-NF35*.

[8] Hassouna M. E. M., Abdelrahman M. M., Mohamed M. A. (2017). Validation of a novel and sensitive RP-HPLC method for simultaneous determination of cefixime trihydrate and sodium benzoate in powder for oral suspension dosage form. *Journal of Forensic Sciences & Criminal Investigation*.

[9] Gowri Sankar D., Nagesh Babu A., Rajeswari A., Vamsi Krishna M. (2009). RP-HPLC method for estimation of flucloxacillin magnesium and sodium benzoate in oral suspension. *Asian Journal of Chemistry*.

[10] The United States Pharmacopoeial Convention (2017). *Betamethasone USP Monograph (USP40-NF35)*.

[11] The United States Pharmacopoeial Convention (2017). *Betamethasone Oral Solution USP Monograph (USP40-NF35)*.

[12] The United States Pharmacopoeial Convention (2017). *Dexchlorpheniramine Maleate USP Monograph (USP40-NF35)*.

[13] The United States Pharmacopoeial Convention (2017). *Dexchlorpheniramine Maleate Oral Solution USP Monograph (USP40-NF35)*.

[14] Mustarichie R., Levitaa J., Musfiroha I. (2014). Spectrophotometric validation method of dexchlorpheniramine maleate and betamethasone. *International Journal of Research and Development in Pharmacy and Life Sciences*.

[15] Fereja T. H., Hymeta A., Bekhit A. A. (2015). Stability indicating HPTLC method development for determination of betamethasone and dexchlorpheniramine maleate in a tablet dosage form. *AASCIT Journal of Chemistry*.

[16] Hoa Le T. H., Phung T. H., Le D. C. (2019). Development and validation of an HPLC method for simultaneous assay of potassium guaiacolsulfonate and sodium benzoate in pediatric syrup. *Journal of Analytical Methods in Chemistry*.

[17] AOAC International (2016). Appendix F: guidelines for standard method performance requirements. *AOAC Official Method of Analysis*.

[18] The International Council for Harmonisation of Technical Requirements for Pharmaceuticals for Human Use (ICH) (2005). *Validation of Analytical Procedures: Text and Methodology Q2 (R1)*.

[19] FDA-Guidance for Industry (2010). *Validation of Analytical Procedures: Definition and Terminology Final Guidance*.

[20] Naseef H., Moqadi R., Qurt M. (2018). Development and validation of an HPLC method for determination of antidiabetic drug alogliptin benzoate in bulk and tablets. *Journal of Analytical Methods in Chemistry*.

[21] Swartz M. E., Krull I. (2006). Method validation and robustness. *LCGC North America*.

